# The Cost of Autism Spectrum Disorders

**DOI:** 10.1371/journal.pone.0106552

**Published:** 2014-09-05

**Authors:** Chiara Horlin, Marita Falkmer, Richard Parsons, Matthew A. Albrecht, Torbjorn Falkmer

**Affiliations:** 1 School of Occupational Therapy & Social Work, CHIRI, Curtin University, Perth, Australia; 2 School of Education and Communication, CHILD Programme, Institute of Disability Research, Jönköping University, Jönköping, Sweden; 3 School of Psychology, CHIRI, Curtin University, Perth, Australia; 4 Rehabilitation Medicine, Department of Medicine and Health Sciences (IMH), Faculty of Health Sciences, Linköping University & Pain and Rehabilitation Centre, Linköping, Sweden; 5 School of Occupational Therapy, La Trobe University, Melbourne, VIC, Australia; Emory University School Of Medicine, United States of America

## Abstract

**Objective:**

A diagnosis of an autism spectrum disorders is usually associated with substantial lifetime costs to an individual, their family and the community. However, there remains an elusive factor in any cost-benefit analysis of ASD diagnosis, namely the cost of *not* obtaining a diagnosis. Given the infeasibility of estimating the costs of a population that, by its nature, is inaccessible, the current study compares expenses between families whose children received a formal ASD diagnosis immediately upon suspecting developmental atypicality and seeking advice, with families that experienced a delay between first suspicion and formal diagnosis.

**Design:**

A register based questionnaire study covering all families with a child with ASD in Western Australia.

**Participants:**

Families with one or more children diagnosed with an ASD, totalling 521 children diagnosed with an ASD; 317 records were able to be included in the final analysis.

**Results:**

The median family cost of ASD was estimated to be AUD $34,900 per annum with almost 90% of the sum ($29,200) due to loss of income from employment. For each additional symptom reported, approximately $1,400 cost for the family per annum was added. While there was little direct influence on costs associated with a delay in the diagnosis, the delay was associated with a modest increase in the number of ASD symptoms, indirectly impacting the cost of ASD.

**Conclusions:**

A delay in diagnosis was associated with an indirect increased financial burden to families. Early and appropriate access to early intervention is known to improve a child's long-term outcomes and reduce lifetime costs to the individual, family and society. Consequently, a per symptom dollar value may assist in allocation of individualised funding amounts for interventions rather than a nominal amount allocated to all children below a certain age, regardless of symptom presentation, as is the case in Western Australia.

## Introduction

A diagnosis of an Autism Spectrum Disorder (ASD) results in an estimated annual national cost to Australia of $4.5–7.2 billion [1] that is borne by the individuals themselves, their families, their community, and by government. Costs of autism can peak during the periods when a diagnosis is being assessed and when treatments are being administered, but many costs are ultimately on-going and constitute a life-long burden. The accurate identification of ASDs necessarily relies on assessing observable behaviours using timely, accurate, reliable and valid diagnostic procedures. At present, the “gold standard” diagnosis of autism is a lengthy and time consuming process that requires a suitably qualified multi-disciplinary team (MDT) to assess behavioural, historical and parental report information to determine a definitive diagnosis [2]–[4]. This is discouraging since failing to accurately identify, or prolonging the identification of, children as having ASD will delay access to apposite intervention and support services [5], [6]. This issue is compounded by the possible 25 fold increase in the recent diagnosis rates of ASD in Australia [1], and resultant pressing demand for delivery of diagnostic services and intervention.

It is possible that the delay in treatment and support for children with ASD results in significant costs. Recently, the estimated annual adjusted costs for an adult and a child with autism have been calculated as follows (all dollar values are in AUD and rounded to the nearest dollar): 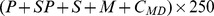
where, *P*  =  production loss of the individual and family with an ASD diagnosis, *SP*  =  support costs, *S*  =  school costs, mainly addition and specialist staff, *M*  =  medical costs, and *C_MD_* =  missed diagnosis. It is important to note that several assumptions are made regarding productivity costs that we do not feel fully encompass the impact of a child's diagnosis on parent employment resulting in what appears to be a very low estimate of the financial impact (e.g., time taken out from current employment for treatment visits is accounted for, but reduced employment of parents so that they can care for a child with ASD is not taken into account). Notably, estimates for the parameter *C_MD_* (missed diagnosis) have not been made and, as a result, the true benefit of a diagnosis of ASD could not be calculated. It seems plausible that an early diagnosis of autism may reduce the cost of ASD because diagnosis leads to early intervention, which results in better outcomes [7], [8], improved social behaviour [9], [10], and less reliance on specialised education support classes [7], [9]. These improvements may, in turn, have knock-on benefits to families and society more broadly, as well as increased productivity for parents and the individual themselves later in life. These gains are usually accomplished when intervention is commenced very early, between the ages of 2 and 4 [11], [12].

However, the complication here is clear: those individuals remaining undiagnosed are, for that very reason, inaccessible to service providers and researchers alike. The next closest approximation that can be made for parameter *C_MD_* is therefore the difference in costs between those children identified and treated early in their development and those not identified and/or treated until later in childhood or even adolescence and adulthood. One method of estimating this cost is to identify a subgroup of children who receive a diagnosis of ASD shortly after their parents suspect their child's development is atypical, and then compare costs for these children with a subgroup of children who had their diagnosis formally confirmed much later. However, one possible offset of the cost of early diagnosis is the long-term accumulated cost of interventions and special services. Therefore, the potential added cost associated with a later diagnosis may not be substantially higher than an earlier diagnosis.

Research into the impact of receiving an early diagnosis and on a family's financial burden is limited [4]. In Australia, state and territory governments are primarily responsible for supporting disability, including ASD, rather than the Commonwealth. However, families with a child diagnosed with an ASD receive a finite amount of Commonwealth funding up to the child's seventh birthday (the so-called “Helping Children with Autism “-funding) for the purposes of early intervention. From this point onwards, ongoing therapies and services outside of school are largely parent-funded on top of other medical and non-medical costs. Aside from the obvious cost to families of missing this early opportunity for government funding, the current study also seeks to determine whether a delayed diagnosis results in inflated costs, reduced incomes and increased financial stresses to families on a long-term basis when compared to families of individuals with a more immediate the immediate diagnosis. Thus it will be possible to estimate an accessible proxy of the parameter *C_MD_*.

## Methods

### Participants and Procedures

With the assistance of the Disabilities Services Commission (DSC) Western Australia, a questionnaire was distributed to families with children registered as having an ASD on their client register. Only families with diagnosed children currently under the age of 18 years were included. This decision was made in line with recommendations from the DSC based on the likelihood of correct/valid information being extractable from the register. At the time of the mail out, 3,965 children were registered with DSC from 3,723 families. Of the packages mailed out, 3,494 were sent to families with one child with ASD, 217 packages sent to families with two diagnosed children, 11 packages sent to families with three diagnosed children and one package sent to a family with four children diagnosed with ASD. Families with more than one child under 18 received one questionnaire for each child with ASD. Of the 3,723 questionnaire packs (covering 3,965 children) sent out by DSC, 192 were returned as “address unknown”. In total, 521 questionnaires were returned, resulting in a response rate of 15%.

#### Questionnaire Development

Development of the full parent-report questionnaire was informed by anecdotal reports from clinical experts and families, the current research literature and insurance reports. Firstly, the general areas of interest were listed and all financial aspects were itemised and categorised to cover as many potential expenses that were a consequence of having a child with ASD. The questionnaire also attempted to gather and summarise information on all possible direct medical, direct non-medical and indirect costs associated with having a child with an ASD. The areas of expense addressed in the questionnaire are listed in Table 1 and specific items relevant to each area are presented in the final questionnaire in the Appendix S1 in the provided Supporting Information.

**Table 1:** Areas of expense for families with children with ASD addressed in the questionnaire.

Direct Medical	Direct Non-Medical	Indirect
Physicians/dentists	Childcare	Caregiver lost productivity
Pharmaceuticals	Respite care	Family quality of life
Therapeutic services/interventions	Home improvement	
Alternative/complimentary therapies	Special education	
Emergency room/hospitals	Support services for other family members	
Home healthcare		
Treatment-related travel		

Questions were then devised to gather the information conveniently and efficiently, which would lead to clear and relevant summary reports whilst taking into account the heterogeneity of families with ASD. A pilot version of the questions and response formats were sent for comments to a number of clinical psychologists, neuropsychologists, developmental psychologists, social workers, occupational therapists, and other clinicians and service providers.

A full version of the questionnaire was then piloted on three families who have children with ASD. Based on their comments and feedback the questionnaire was adjusted to its final version (Appendix S1) and comprised a total of 73 items on demographic information, the child's diagnosis, developmental history, treatment history, education, child care and qualitative questions about the child and families' quality of life.

The final page of the questionnaire included a diagnostic checklist of DSM-IV-TR/ICD-10 items [13]. The checklist consists of 20 symptom characteristics divided into 4 sections addressing the traditional symptom domains of;

-Impairments in social interaction (five symptom characteristics),
-Impairments in communication (seven symptom characteristics),
-Restricted, repetitive and stereotyped patterns of behaviour, interest or activities (seven symptom characteristics),
-The presence of impairments in at least one of the above before the age of three (one characteristic).

Parents were asked to indicate whether any of these characteristics currently previously applied to their child.

#### Data Collection

Questionnaire packs were prepared by the authors before being delivered to the DSC. To maintain confidentiality of the DSC's client register, all printing of cover letters and addressing of envelopes was handled by the DSC's staff and at no time did any of the researchers have access to this register. As per the DSC's records, packs were sent to the primary contact, the default for which was the diagnosed child's father. The questionnaire pack included the questionnaire itself and a reply paid return envelope. All questionnaires were de-identified and asked for no identifying information.

After a period of one month, reminders to complete the questionnaire were published online, in newspapers, DSC newsletters and on community radio. Upon receiving deidentified and completed questionnaires, a unique anonymous identifier was allocated to each questionnaire. Responses were then entered into a data file using IBM SPSS version 20 and analysed using the SAS version 9.2 statistical software.

### Data analysis

#### Treatment-related travel costs

Direct costs associated with travel for medical, therapeutic and complimentary/alternative treatments were calculated as a function of the reported frequency of average visits per month (questions 19, 28 and 36 respectively) and distance from these services (questions 18, 27 and 35 respectively). Round trip costs were calculated based on doubling the median kilometre distance from services and the average per kilometre cost of running a small car (approximated at $0.65AUD per kilometre by the most recent Royal Automobile Club figures).

Individual cost estimates were calculated for medical visits relating to a child's ASD diagnosis, therapeutic visits relating to an ASD diagnosis and complimentary/alternative visits undertaken as part of ASD treatment. These were summed to create a monthly cumulative total, which was then adjusted to create an annual estimate of treatment related travel costs according to the following formula: 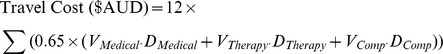
where *V* equals the number of visits per month, *D* equals the distance in km to the service. ‘Medical’, ‘Therapy’ and ‘Comp’ refer to medical professional services, other therapeutic services and complementary and alternative services respectively.

#### Out of pocket treatment costs

The reported annual out-of-pocket costs to families in relation to ASD- specific medical, therapeutic and complimentary/alternative treatment services (questions 22, 33 and 37) were summed to create a total direct treatment cost variable. All three questions had seven potential response options representing intervals of dollar amounts. These options were then converted to the mid-point of each interval to create a single dollar amount for analysis. These recoded variables were then summed to create a cumulative total of the out of pocket costs associated with ASD-specific medical, therapeutic and complimentary/alternative treatments.

#### Loss of income from employment reduction

The productivity loss associated with having a child diagnosed with ASD was calculated based on self-reported impact of their child's diagnosis on the parent/caregiver ability to work (question 51) and, if employment status was affected, the size of this reduction as a function of hours in the average work week (question 52). Those responding to question 51 by selecting options one, two or three, as presented in Table 2, then indicated the reduction in hours in question 52. This reduction was thereafter converted to a proportion of full-time equivalent (FTE) employment. Those indicating that either one or both parents could not work at all at this time due to the needs of their ASD child were converted to an FTE reduction of 1 or 2 units, respectively. Parents indicating that their employment status was unaffected were coded as 0. This variable was then multiplied with the median full-time income for 2010–2011 as reported by the Australian Taxation Office (the latest figures made available), which was $48, 864.

**Table 2:** Questions 51 and 52 and the coding of responses used to estimate proportions of FTE reductions.

*Question 51: How much has your child's diagnosis affected the employment status of your household?*		
1	Both parents must work less hours	
2	One parent (of a two-parent household) must work less hours	
3	Single parent must work less hours	
4	One parent cannot work at this time	**1** FTE reduction
5	Both parents cannot work at this time	**2** FTE reduction
6	Unaffected	**0** FTE reduction
*Question 52: If your employment status has been affected, please estimate by how many hours in an average week your employment load has been reduced.*		
−99	Not relevant	**0** FTE reduction
1	<7 hours	**.2** FTE reduction
2	7–14 hours	**.4** FTE reduction
3	15–21 hours	**.6** FTE reduction
4	22–28 hours	**.8** FTE reduction
5	29–35+hours	**1** FTE reduction

#### Cumulative cost of having a child diagnosed with ASD

To create a single estimate of direct and indirect expenses to each family that were specifically related to having a child diagnosed with ASD, the costs associated with treatment-related travel, out-of-pocket treatment expenses and loss of income/productivity were summed.

#### ‘Immediate’ versus ‘delayed’ diagnosis

A dichotomous ‘immediate’ versus ‘delayed’ diagnosis variable was created based on cross-tabulations between questions 10 (age at first suspicion of something not being quite right) and 12 (age at which a formal diagnosis was received). This variable was intended to capture the chronological difference between those receiving early or more immediate diagnosis (and presumably treatment and intervention) and a later or delayed diagnosis (often after the optimal developmental window for treatment and period at which early intervention would take place). Those children with a zero or one step chronological difference between questions 10 and 12 were coded as ‘immediate’ and those with a two or more step difference between these questions were coded as ‘delayed’. A further, stricter, division of ‘immediate’ and ‘delayed’ was also conducted by retaining only those with a zero step difference between questions 10 and 12 (‘immediate’) and those with a three or more step difference (‘delayed’). These divisions are delineated in Table 3.

**Table 3:** Division of children receiving ‘immediate’ (N = 250) versus ‘delayed’ (N = 266) diagnoses.

	Q12: How old was your child when she/he was formally diagnosed with an ASD?					
		12–18 months*(2)*	19–24 months*(3)*	2–6 years*(4)*	6–12 years*(5)*	13–18 years*(6)*
Q10; How old	<12 months*(1)*	6	15	***65***	***34***	***6***
was your child	12–18 months*(2)*	***5***	22	91	***28***	***2***
when you or	19–24 months*(3)*	-	***7***	74	19	***3***
someone else noticed	2–6 years*(4)*	-	-	***93***	32	3
something was different	6–12 years*(5)*	-	-	-	***10***	-
or not quite right?	13–18 years*(6)*	-	-	-	-	***1***

Numbers in bold indicate the stricter division of ‘immediate’ (N = 116) versus ‘delayed’ (N = 138).

#### Cumulative presence of ASD symptoms

The total number of ASD symptoms was estimated by a cumulative total of the positive indications from the presence of symptoms within the four domains listed on the DSM-IV/TR-ICD-10 checklist. Positive indications were coded as one and responses in domain one to three were summed to create a total for each child. The fourth domain comprised one single developmental history question and was excluded from this total, since this characteristic was regarded as a prerequisite for receiving a diagnosis of ASD.

#### Analysis of non-respondents

Six months after the initial mail-out, a random sample of 405 families registered with the DSC were contacted for a telephone follow-up. Given the confidentiality of the DSC register and the de-identified nature of the returned questionnaires, it was not known which families on the register had or had not completed the long-form questionnaire. Thus, the random sample of 405 families would include those that had completed and returned a questionnaire and those that had not. Non-respondents were then asked to answer an abbreviated form of the questionnaire over the phone. This short-form phone questionnaire consisted of twenty questions taken from the original questionnaire for the purposes of a later drop-out analysis. Those questions included in the short-form are shown in italics in Appendix S1. For the purposes of comparisons between those that did and did not respond to the long-form questionnaire that was sent out via mail, independent samples *t-*tests were used to compare the ages of children, chi-square tests were used to compare categorical demographic variables and Mann-Whitney U tests were conducted to compare calculated cost variables. Due to the shortened nature of the short-form questionnaire, only the out of pocket treatment costs and the loss of income from employment reduction variables could be calculated for both respondents and non-respondents. The response categories of some demographic variables (question 10 and 12 specifically) were collapsed to ensure validity of the chi-square test.

### Ethical approval

Ethical approval was obtained from the Curtin University Human Research Ethics Committee (HR 138/2012) and the internal ethical review board of the DSC in Western Australia. Questionnaire packs were sent to the DSC's clients with a cover letter from the Director General of DSC explaining the nature and purpose of the study as well as an information sheet inviting families to complete and return the questionnaire. Completed and returned questionnaires were taken as consent to participate in the study.

## Results

The characteristics of the included 521 children with ASD are presented in Table 4. Descriptive statistics for all cumulative cost estimates and the cumulative presence of ASD symptoms are presented in Table 5. An additional total cost of ASD variable for families with only one child diagnosed with ASD was calculated to express the costs of ASD without the compounding effect of having multiple children with ASD and the implications this may have to reductions in FTE employment. The cumulative ‘Cost of ASD’ variable predominantly consisted of loss of income of the parents and caregivers (89%) with ASD-related travel costs (3%) and treatment costs (8%) making smaller contributions.

**Table 4:** Characteristics of the children with ASD and their families.

	N		%	
**Total**	521			
Male	431		83	
Female	90		17	
**Age (months)**	**Mean** (SD) 119 (50)		**Median** 113.5	
**Respondent**				
Biological mother	421		81	
Biological father	87		17	
Grandparent	5		.96	
Foster parent	4		.77	
Step parent	1		.2	
Other	1		.2	
**ASD diagnosis**				
Autism	272		52.60	
High-functioning autism	128		24.70	
Asperger Syndrome	36		7	
PDD-NOS	76		14.70	
CDD	2		.40	
Other	7		1.40	
**How many children with ASD have one or more ASD sibling?**				
0 (only child with ASD)	355		71	
1 sibling	121		24	
2 siblings	15		3	
3 siblings	9		2	
**Presence of CD/ID**				
Yes	371		72	
No	144		28	
**Presence of other mental health/psychological conditions**				
Yes	408		79	
No	108		21	
**Presence of other medical conditions**				
Yes	341		66.70	
No	170		33.30	
**How old was your child when you first noticed something wasn't right?**				
<12 months	126		24	
12–18 months	150		29	
19–24 months	103		20	
2–6 years	128		24.70	
6–12 years	10		2	
13–18 years	2		.3	
**How old was your child when formally diagnosed?**				
<12 months	0		0	
12–18 months	11		2.10	
19–24 months	44		8.50	
2–6 years	325		62.70	
6–12 years	123		23.70	
13–18 years	15		2.90	
**Parent's highest education**	**N**	**%**	**N**	**%**
	Mother		Father	
Completed year 10	72	14	74	15
Completed year 12	60	12	42	8.5
Completed certificate at TAFE (or similar)	135	26.40	87	17.60
Apprenticeship	13	2.50	94	19
Some university education but did not complete	46	9	36	7.60
Completed university undergraduate degree	103	20.10	85	17.20
Completed university postgraduate degree	82	16	75	15.20
**Household composition**	**N**		**%**	
Two-parent	396		76.90	
Single parent	72		14	
Only extended family (grandparents etc)	4		.80	
Two-parent plus extended	22		4.30	
Single parent plus extended	10		2	
Foster situation	3		.60	
Other	8		1.50	
**Combined annual income**				
<$25,000	55		10.50	
$25,000–$50,000	56		10.70	
$50,000–$75,000	68		13	
$75,000–$100,000	90		17.30	
$100,000–$125,000	59		11.30	
$125,000–$150,000	65		12.50	
$150,000–$200,000	57		11	
>$200,000	50		9.60	
Unknown	21		4	

Percentage values are rounded to two decimal places where possible.

**Table 5:** Descriptive statistics for all estimated cost variables (rounded to nearest dollar) and cumulative presence of ASD symptomatology.

	N	Mean (SD)	Median	Quartiles		
				1^st^	2^nd^	3^rd^
ASD-related travel	521	$1,500 ($1,200)	$860	$620	$860	$2,000
Out of pocket treatment	370	$4,800 ($5,000)	$2,600	$1,000	$2,600	$7,500
Loss of income	474	$30,000 ($20,300)	$29,200	$19,500	$29,200	$48,700
**Cost of ASD**	**339**	**$37,60 ($21,700)**	**$37,800**	**$22,000**	**$37,800**	**$52,800**
**Cost of ASD (1 ASD child per household)**	**223**	**$35,100 ($20,300)**	**$34,900**	**$20,700**	**$34,900**	**$51,700**
Frequency of ASD symptoms	508	12.4 (4.2)	13	10	13	16

### The cost of ASD as a function of ‘immediate’ versus ‘delayed’ diagnosis or frequency of ASD symptoms

A regression analysis was conducted with cost of ASD as the dependent variable. Independent variables included: ‘immediate’ (coded as 1) versus ‘delayed’ (coded as 2) diagnosis, ASD symptom frequency (as continuous numeric), age of child in years, number of siblings (0, 1, 2 or 3+), number of siblings with ASD (0, 1, or 2+), combined income (in $AUD), age of diagnosis of child with autism (1 = 0–12 months, 2 = 12–24 months, 3 = 2–6 years, 4 = 6–12 years, 5 = 12–18 years) and highest education of the mother and father (1 = year 10, 2 = year 12, 3 = TAFE, 4 = Apprenticeship, 5 = some university, 6 = Bachelor degree, 7 = postgraduate degree). The residuals from the regression analysis were plotted (histogram and density plots) and followed a normal distribution. The R^2^ values for the two models evaluating the different criteria for immediate versus delayed diagnosis (i.e., loose and strict cut off) below were 21% and 31%, respectively, as shown in Table 6.

**Table 6:** Regression model of symptom frequency and delay from first identifying a problem to ultimate diagnosis and the total cost including covariates.

	Full available sample (final N = 332)						Strict immediate vs delayed sub-sample (final N = 152)					
	df	*F*	*p^a^*	Contrast	Coefficient	95% CI	df	*F*	*p^a^*	Contrast	Coefficient	95% CI
Immediate/Delayed diagnosis	1	0.13	0.72	1 vs 2	$-1500	−6000, 3100^b^	1	0.17	0.68	1 vs 2	$2100	−6200, 10500^b^
**Cumulative symptom presence**	**1**	**33.0**	**<0.0001**	**slope**	**$1400^c^**	**860, 1900**	**1**	**15.36**	**<0.0001**	**slope**	**$1500^c^**	**630, 2400**
**Age group**	**4**	**2.77**	**0.028**	**1 vs 5**	**$730**	**−15000, 16000**	4	1.58	0.18	1 vs 5	$-2200	−28300, 23900
				**2 vs 5**	**$720**	**−15000, 16000**				2 vs 5	$-80	−25300, 25200
				**3 vs 5**	**$-340**	**−16000, 15000**				3 vs 5	$-1900	−27400, 23600
				**4 vs 5**	**$-6900**	**−22000, 8300**				4 vs 5	$-9100	−34200, 15900
Number of Siblings	3	2.33	0.075	0 vs 3+	$4300	−4700, 13200	3	1.88	0.14	0 vs 3+	$15600	685, 30600
				1 vs 3+	$-4000	−11300, 3100				1 vs 3+	$2100	−8800, 13000
				2 vs 3+	$-4800	−12300, 2700				2 vs 3+	$1600	−9400, 12700
**Number of Siblings with ASD**	**2**	**5.34**	**0.005**	**0 vs 2+**	**$-29500**	**−46000, −13000**	**1**	**5.24**	**0.007**	**0 vs 2+**	**$-44500**	**−71700, −17400**
				**1 vs 2+**	**$-24600**	**−35600, −13600**				**1 vs 2+**	**$-35300**	**−52100, −18500**
Income	1	2.24	0.14	slope	$-800	−1900, 290	1	3.34	0.07	slope	$-1400	−3100, 270
Age of Diagnosis	4	0.18	0.95	1 vs 5	$9000	−12800, 30800	4	0.26	0.90	1 vs 5	$18800	−14100, 51800
				2 vs 5	$5300	−10800, 21500				2 vs 5	$12400	−15200, 39900
				3 vs 5	$5600	−9100, 20300				3 vs 5	$11200	−9500, 31900
				4 vs 5	$3900	−11300, 19100				4 vs 5	$9400	−11500, 30200
Education (Max of M/D)	7	0.31	0.95	1 vs 8	$17700	−5700, 41200	7	0.41	0.90	1 vs 8	$21600	−39300, 46000
				2 vs 8	$10300	−1100, 21700				2 vs 8	$8200	−5300, 27000
				3 vs 8	$1300	−7500, 10100				3 vs 8	$8800	−24200, 10500
				4 vs 8	$2800	−4300, 9800				4 vs 8	$5500	−12200, 9700
				5 vs 8	$2100	−5200, 9400				5 vs 8	$5900	−16500, 7000
				6 vs 8	$1400	−7200, 10000				6 vs 8	$7600	−15800, 14300
				7 vs 8	$1700	−4500, 8000				7 vs 8	$4900	−13300, 5900

a The p-value was obtained from a regression model using the square root of the total cost as dependent variable (because of skewness in this variable).

b This is the CI for the difference in cost between early and late.

c This is the amount by which the cost increases (dollars) per unit increase on the symptom score.

Regardless of whether the strict or loose definition of the ‘immediate ’versus ‘delayed’ diagnosis variable was used, neither was found to be significantly associated with the of cost of ASD. However, in both regression models, the number of ASD symptoms present was a significant predictor. In both models, costs were increased by approximately $1,400 per ASD symptom reported for the loose and strict criteria models. Models with fewer covariates were also fitted yielding similar estimates for the cost of immediate versus delayed diagnosis and the cumulative presence of ASD symptoms.

### Mediation analysis

A delay in diagnosis and treatment may have an indirect effect on costs associated with ASD by increasing the number of symptoms present. In order to test this hypothesis, a mediation analysis was conducted. Firstly, it was confirmed that a delay in diagnosis was statistically significantly associated with an increased number of symptoms (number of increased symptoms associated with delay  = 1.56, p = 0.001, 95% CI = 0.63, 2.49). Following this, two models were constructed and contrasted: 1) total costs were modelled as a function of delay in diagnosis alone, with the direct effect of diagnostic delay denoted as *c*, and 2) total costs were modelled as a function of delay in diagnosis and cumulative symptom count, with the parameter estimate for delay in diagnosis with the effect attributable to cumulative symptom count partialled out denoted *c′*. The final mediated effect was calculated by subtracting *c′* from *c* (i.e., mediated effect  = *c – c′*). To produce 95% CIs around the mediated effect, 10,000 bootstrapped samples were taken and the quantiles representing 2.5% and 97.5% of the *c - c′* distribution calculated. Bootstrapping the estimate of the mediated effect has been recommended for mediation analysis because the technique offers acceptable statistical power properties, directly estimates the mediated effect, and does not rely on many of the assumptions necessary for other tests [14], [15].

The mediation analysis was supportive of this hypothesis. A statistically significant reduction in the effect associated with diagnostic delay of $2,110 (95% CI = 820, 3700) was observed when symptom count was included in the model. This was despite the delay in diagnosis not being statistically significantly associated with total cost directly (see above). The mediation analysis was repeated with all covariates included in the original model (age of child, number of siblings, number of siblings with ASD, combined income, age of ASD diagnosis, and highest education of mother and father) and a similar effect was found (mediated effect *c-c′* = $1,770, 95% CI = 360, 3340).

### Analysis of non-respondents

**Figure 1:**
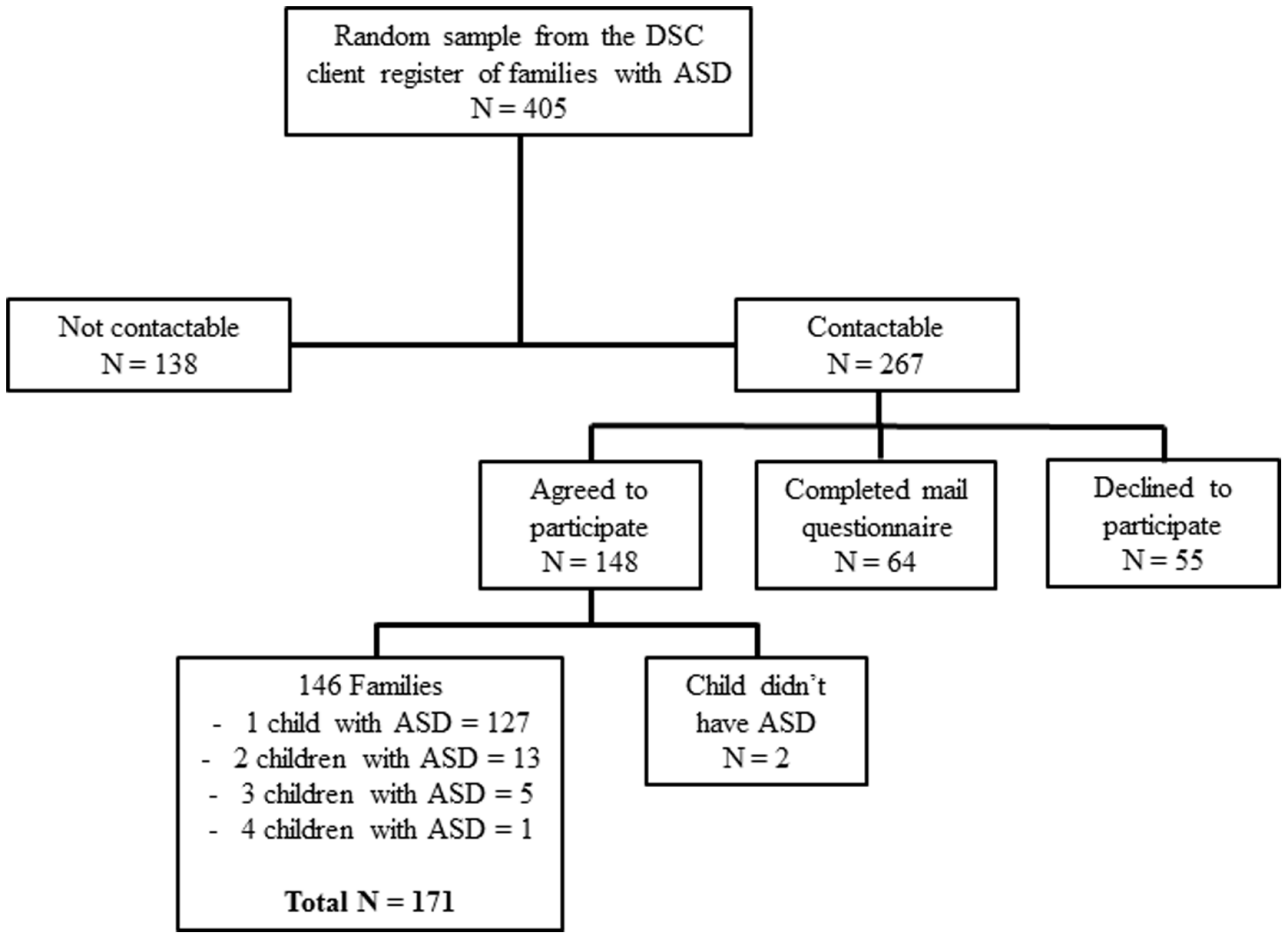
Breakdown of the non-respondent sample for the purposes of the short-form telephone questionnaire. A full representation of a random sample of families registered as having received or currently receiving service provision for the purposes of an analysis of non-respondents. From a random sample of 405 families, 146 families (totalling 171 children with ASD) agreed to participate in the telephone questionnaire

Contact was established with 267 of the 405 families who did not return a completed questionnaire, and, of these, 148 agreed to complete the short-form questionnaire. Sixty-four families had already completed the long-form questionnaire. Fifty-two families (13%) declined to participate in the phone questionnaire. Incorrect phone numbers and no answer were the main reasons for failures to contact. A complete breakdown of the telephone sample is shown in Figure 1. Two of the 148 families that agreed to participate did not have a child with ASD. From the 146 families with children with ASD that agreed to complete the short- form questionnaire, data were collated for 171 individual children.

As shown in Table 7, there were few noteworthy differences between respondents and non- respondents. Demographically, respondents were slightly more likely to have a male child and to have noticed developmental atypicality earlier. Respondents also received a formal diagnosis of ASD earlier than non-respondents. Respondents reported higher treatment costs than non-respondents. However, there were no differences in reported income loss.

**Table 7:** Comparison between respondents (N = 521) and non-respondents (N = 171) on demographics and two main study variables that can be derived from the short-form telephone questionnaire.

	Non-Respondents (N = 171)	Respondents (N = 521)
**Age (months)**	Mean (SD) 122 (50)	Mean (SD) 119 (50)
	Median 120	Median 113.50
		*t* (678) = .53, *p* = .60
	**Proportion (%)**	**Proportion (%)**
**Sex of diagnosed child**		
Male	75.50	83
Female	24.50	17
		*χ^2^*(1, *N* = 689) = 4.41, *p* = .04
**ASD diagnosis**		
Autism	47.40	52.60
HFA	25.10	24.70
Asperger Syndrome	11.10	7
PDD-NOS	15.80	14.70
CDD	.60	.40
Other	-	1.40
		*χ^2^*(6, *N* = 703) = 4.31,*p* = .64
**How old was your child when you first noticed something was not right?**		
<12 months	24.10	24
12–18 months	17.10	29
19–24 months	18.20	20
2–6 years	35.30	24.70
6–18 years*	5.30	2.30
		*χ^2^*(4, *N* = 704) = 15.38,*p*<.05
**How old was your child when formally diagnosed?**		
<12–24 months*	10.30	10.60
2–6 years	50.10	62.70
6–18+ years*	38.70	26.60
		*χ^2^*(2, *N* = 698) = 9.61, *p*<.01
**Household composition**		
Two-parent	73.70	76.90
Single parent	22.20	14
Only extended family (grandparents etc)	.60	.80
Two-parent plus extended	2.30	4.3
Single parent plus extended	1.20	2
Foster situation	-	.6
Other	-	1.5
-		*χ^2^*(6, *N* = 702) = 10.94, *p* = .09
***Out-of-pocket treatment costs***	Mean (SD) $2,300 ($2,900)	Mean (SD) $4,800 ($5,000)
-	Median $1,000	Median $2,600
-		*Z* = −16.95, *p*<.001, *r* = −.76
***Loss of income***	Mean (SD) $25,400 ($21,000)	Mean (SD) $30,000 ($20,300)
-	Median $29,300	Median $29,200
-		*Z = −.94*, *p* = .35, *r* = −.04

Categorical demographic variables are presented as proportions due to missing data in some variables.

^*^ These variables have been collapsed across categories for the purposes of chi-square analyses.

## Discussion

ASD-related costs were strongly associated with the cumulative presence of the child's symptoms. This builds upon previous research that has found that having a child with ASD is associated with significant financial strain [16]–[19]. The association between increased costs and ASD symptom severity suggests that effective and early interventions that result in the reduction of expressed symptoms may have a significant impact on improving a family's productivity and their resultant financial situation.

Contrary to expectations, there was no statistically significant differences in costs related to receiving the diagnosis of ASD whether soon or late after suspicion. We suggest two possible reasons for this. Firstly, given the above finding that increasing symptomatology is directly related to the cost of ASD, the more immediate identification of ASD may create a situation where improvement in outcomes and the effect on associated symptomatology measures dwarfs the influence of delay to diagnosis on the final cost of ASD. This interpretation appears to be consistent with the mediation analysis, whereby an indirect effect of diagnostic delay on costs appeared to be mediated by an association between increased symptomatology and increased diagnostic delay. Secondly, families with a child with ASD may adapt their work-family balance regardless of whether the child has a diagnosis or not. This is reflected by the finding that the largest cost reported by parents was a loss of income from reduced working hours. This is consistent with a previous report stating that a loss of approximately 14% of family income is often the consequence of having a child with ASD [16], equalling $7,200 (based on the median income and average exchange rate of 2008). The results from the present study suggest a substantially higher (29%) loss of combined household income.

From a family and societal perspective, support that allows family members to work may more effectively assist families with children with ASD by lessening the financial burden and improving well-being of all family members. Furthermore, the long-term consequence of high costs associated with having a child who has a diagnosis of ASD is not well researched. A study reporting on the distribution of societal costs of ASD distributed throughout the lifespan estimated that fathers of children with moderate to severe autism were unemployed 20% of a full time equivalent [20], whereas 60% of mothers were unemployed and 30% worked part time of a full time equivalent [20]. If these estimates are indeed accurate, the family income may therefore be severely impacted through the lifespan as a result of having a child with ASD [20]. The largest direct life time cost is to provide care for adults with ASD [20], [21]. Consequently, parents of adults with ASD may face an even larger financial strain when having to provide services for their adult children whilst having had less opportunity to save or accumulate superannuation funds due to reduced possibilities to work full time [20].

### Limitations

There are several limitations to the cost analysis presented here: 1) The return rate of 15% observed in the present study is quite poor in comparison to similar studies. This may be because client details registered with the DSC are maintained sporadically and a number of records contained incomplete or inaccurate entries. Furthermore, distributed questionnaires were addressed only to the fathers of the registered child for some families due to a DSC database error. Given reports of higher than normal divorce rates in families with children with ASD [22], this may be another reason for a lower than usual return rate reflected by the higher than expected number of respondents reporting being in two-parent households compared to non-respondents. 2) There are multiple reasons why a child might not be diagnosed until later in development, giving rise to a number of caveats with respect to the ‘delayed’ diagnostic group. For example, some of those diagnosed later in development may present with symptomatology that is either more complex or reduced in severity compared to those children with evident and more severe symptoms from an early age. However, this influence appeared to be relatively small as the effect of the variable “Age of diagnosis” was not a statistically significant contributor to cost, or may be reflected more by the cumulative presence of ASD symptomatology measure. 3) Requesting estimates of current costs may not be representative of historical costs as current expenses are dependent on the child's stage of development, time since diagnosis, and current stage of treatment or intervention. However, one aspect for which our estimates are robust is their specification of only ASD-related expenses for medical, behavioural or complimentary/alternative therapies. 4) In contrast to most parents that had reduced their hours due to the needs of their child with ASD, a small number of parents were seeking *more* work due to the expenses of having a child with ASD. This is not often considered when calculating the costs and productivity losses associated with a diagnosis of ASD. 5) A number of parents reported low treatment costs because they had exhausted all funded avenues and could not afford to independently fund treatment for their children. 5) Lastly, the present study does not address all aspects of how an immediate or delayed diagnosis may impact families and their financial situation. The processes that parents undergo in order to access a proficient diagnosis are reported as being extremely stressful [23] and may result in health related consequences difficult to estimate in any cost benefit analysis [18]. Consequently, health related consequences and costs for family members and society in relation to early/late diagnosis may warrant further scrutinising.

## Conclusions

The median family cost of ASD was estimated to be AUD $34,900 per annum (IQR $20,700 - $51,700; based on median income from wages), with almost 90% of the sum ($29,200) due to loss of income from employment. While there was no statistically significant direct effect on the cost depending on the timeliness of the diagnosis (immediate versus delayed) the cumulative presence of ASD symptoms had a significant impact on the costs and a delay in diagnosis could indirectly increase costs by neglecting symptoms that may respond to more immediate intervention. For each additional symptom reported, a $1,400 cost for the family per annum was added. These findings take on a great deal of significance when considered within a regional context as funding amounts vary across states and countries. The financial burden of families of children with ASD is correlated with the existing societal financial safety net [17]. In an Australian context, families may miss out on the most widely available financial support for early intervention if the diagnosis is received after the age of six [24]. If the family is expected to carry a substantial share of the cost needed to support the development of children with ASD, this may have detrimental consequences for the wellbeing of the child with ASD, as well as for other family members, especially for low income families that may not seek services at all for financial reasons [17].

## Supporting Information

Appendix S1 **The complete questionnaire.** Those questions included in the short form are shown in italics.
(DOCX)
