# Supplementary material for: The Cost of Autism Spectrum Disorders
Source: PLoS One. 2014 Sep 5;9(9):e106552. doi: 10.1371/journal.pone.0106552 (PMC4156354; doi:10.1371/journal.pone.0106552)
Supplement: Appendix S1 — The complete questionnaire. Those questions included in the short form are shown in italics. (DOCX) [file pone.0106552.s001.docx]

Appendix S1

Experiences and Expenses of Families with a Child Diagnosed with an Autism Spectrum Disorder

The following are a series of questions regarding your life and experience as a parent/family with a young, adolescent or adult child that has received a diagnosis of an Autism Spectrum Disorder (ASD). We are particularly interested in understanding the financial pressures experienced by families with children diagnosed with an ASD and the process you went through to access a diagnosis and intervention services.

As the primary caregiver of a child diagnosed with an ASD, please answer these questions with the best or closest estimate that you can or select the response that is closest to your situation.

Some questions may include a section for a written response if you wish to do so.

Questions with an ***** are specific to parents with an adolescent or adult child with an ASD and may not be applicable to those with young children.

Please do not hesitate to contact the researchers if you have any questions or queries regarding this questionnaire.

**ABOUTYOUR CHILD/CHILDREN**

1. *What is the gender of your child diagnosed with ASD?*

- Male
- Female

2. *What is your diagnosed child's current age (in years and months; e.g. 2 years 4 months)?*

3. *What is your relationship to the child diagnosed with an ASD?*

- Biological Mother
- Biological Father
- Grandparent
- Foster Parent
- Step Parent
- Other

4. *How many biological children do you have?*

- 0
- 1
- 2
- 3
- 4
- More than 4

*5. How many of your other biological children have a diagnosis of an Autism Spectrum Disorder?*

- 0
- 1
- 2
- 3
- More than 3

6. *What is your child's official ASD diagnosis?*

- Autistic Disorder (Autism)
- High-functioning autism
- Asperger’s Syndrome
- Pervasive Developmental Disorder – not otherwise specified (PDD-NOS)
- Rett’s Syndrome
- Childhood Disintegrative Disorder
- Other

7. *Does your child with ASD also have a diagnosis of cognitive impairment/intellectual disability or similar?*

- No
- Yes (please specify)

8. *Does your child with ASD have any other diagnosed psychological/mental health conditions?*

- No
- Yes (please specify)

9. *Does your child with ASD have any other physical or medical conditions or diagnoses?*

- No
- Yes (please specify)

10. *How old was your child when you or someone else first noticed something was different or not quite right?*

- Less than 12 months
- 12 - 18 months
- 19 - 24 months
- 2 – 6 years
- 6 - 12 years
- 13 - 18 years
- 18+ years

Please specify what that behaviour was _______________________________________________________________________________

_______________________________________________________________________________

11. How old was your child when you first sought advice about your concerns?

- Less than 12 months
- 12 - 18 months
- 19 - 24 months
- 2 – 6 years
- 6 - 12 years
- 13 - 18 years
- 18+ years

12. *How old was your child when she/he was formally diagnosed with an ASD?*

- Less than 12 months
- 12 - 18 months
- 19 - 24 months
- 2 – 6 years
- 6 - 12 years
- 13 - 18 years
- 18+ years

13. Who diagnosed your child?

- Medical doctor/Paediatrician
- Psychiatrist
- State/territory government assessment service/multidisciplinary team
- A private multidisciplinary team/service
- Other (please specify)

14. What was your first reaction to your child's diagnosis of ASD?

- Agreed with the diagnosis
- Disagreed with the diagnosis but did not seek a second opinion
- Disagreed with the diagnosis and sought a second opinion
- Other (please specify)

**ABOUT YOUR ACCESS TO MEDICAL AND NON-MEDICAL SERVICES AND COSTS RELATED TO DIAGNOSIS AND TREATMENT**

15. What type of insurance coverage does/did your child with ASD have?

- Private health insurance
- Medicare but no private insurance
- Medicare and private insurance
- None

16. If you have/had private health insurance, how do/did you pay for claimable medical/behavioural/diagnostic services for your child with ASD?

- Medicare
- Private health insurance

17. *****What type of insurance coverage does your adult son/daughter with ASD have?

- Private health insurance
- Medicare but no private insurance
- Medicare and private insurance
- None
- Unknown

18. How close are you to your MEDICAL facility (G.P., paediatrician etc) or, how close does your adult child diagnosed with ASD live with respect to medical services that he/she requires?

- Less than 2 kilometres
- 2-5 kilometres
- 6-10 kilometres
- 11-20 kilometres
- 21-30 kilometres
- Greater than 30 kilometres

19. How many ASD-related MEDICAL visits/appointments does your child have in an average month? NB: ASD-related medical visits are those visits only related to aspects of your child’s ASD diagnosis i.e. medications etc.

- Less than 2
- 2
- 3
- 4
- 5
- 6
- 7
- 8 or more

20. How many non-ASD-related MEDICAL visits/appointments does your child have in an average month? NB: non-ASD related visits are those related to other chronic medical conditions or general illness

- Less than 2
- 2
- 3
- 4
- 5
- 6
- 7
- 8 or more

21. What is the total cost (what you pay plus what insurance/Medicare pays) of your diagnosed child's ASD-related MEDICAL treatment (doctors’ visits, medications etc.) per year? (Please estimate)

- Less than $500
- $500 – 2,000
- $2,001 – 6,000
- More than $6,000
- Unknown

22. *How much of the total cost of ASD-related MEDICAL treatment (doctors’ visits, medications etc.) do you pay "out of pocket" per year? (On average)*

- *Less than $250*
- *$250 - 500*
- *$501 – 1,000*
- *$1,001 – 2,000*
- *$2,001 - 4,000*
- *$4,001 - 6,000*
- *More than $6,000*
- *Unknown*

23. Did you claim on Medicare for the ASD diagnosis or development of an ASD treatment/management plan for your child by a paediatrician/psychiatrist (before 13 years of age)?

- Yes
- No
- I cannot remember

24. Please indicate which of the following Medicare-claimable services collaborated with the paediatrician/psychiatrist on the ASD diagnosis of your child. Please tick/mark all that may apply to you.

- Audiologist
- Occupational therapist
- Optometrist
- Orthoptist
- Physiotherapist
- Psychologist
- Speech Pathologist
- None
- Other (please specify):

25. Please indicate which of the following Medicare-claimable services you accessed for intervention treatment services after the diagnosis of your child

- Audiologist
- Occupational therapy
- Optometrist
- Orthoptist
- Physiotherapist
- Psychologist
- Speech Pathologist
- None
- Other (please specify):

26. If you have private health insurance, please specify what services your insurance covers (select all that apply).

- Medication
- Diet
- Audiology
- Occupational therapy
- Optometry/Orthopty
- Physiotherapy
- Psychologist
- Psychiatrist
- Speech Pathologist
- Respite
- Other (please specify):

27. How close are you (on average) to your THERAPEUTIC services (those listed in questions 25 & 26) or, how close does your adult child diagnosed with ASD live with respect to these services?

- Less than 2 kilometres
- 2-5 kilometres
- 6-10 kilometres
- 11-20 kilometres
- 21-30 kilometres
- Greater than 30 kilometres

28. How many THERAPEUTIC (any non-medical service listed above) visits/appointments not related to intervention or behavioural therapy does your child have in an average month?

- Fewer than 2
- 2
- 3
- 4
- 5
- 6
- 7
- 8 or more

29. If your child took part in, or is taking part in, Early Intervention therapy (ASD intervention prior to 7 years), how many hours did/do they complete in an average week?

- 2 or less
- 3 – 6 hours
- 7 - 10 hours
- 11 - 14 hours
- 15 – 18 hours
- 18 – 20 hours
- More than 20 hours
- Not Applicable

30. If your child IS OLDER THAN 7 YEARS, how many hours of intervention/behavioural therapy do they complete in an average week as part of their ASD treatment?

- 2 or less
- 3 – 6 hours
- 7 - 10 hours
- 11 - 14 hours
- 15 – 18 hours
- 18 – 20 hours
- More than 20 hours
- Not Applicable

31. Has ASD-related intervention/behavioural therapy improved your child’s quality of life?

- Yes, definitely
- Yes, somewhat
- Neutral
- Disagree
- Strongly Disagree
- Not Applicable

32. What is the total cost (what you pay plus what Medicare/insurance pays) of your child's ASD-related THERAPEUTIC treatment (services listed in questions 25 & 26) per year? Please estimate.

- Less than $500
- $500 – 2,000
- $2,001 – 6,000
- More than $6,000

33. *How much of the total cost of ASD-related THERAPEUTIC treatment (the claimable services listed above) do you pay "out of pocket" per year (on average)? Please estimate.*

- *Less than $250*
- *$250 - 500*
- *$501 – 1,000*
- *$1,001 – 2,000*
- *$2,001 - 4,000*
- *$4,001 - 6,000*
- *More than $6,000*

34. Which of the following COMPLEMENTARY/ALTERNATIVE (non-claimable) services do you access to aid the treatment of your child’s ASD?

| ALTERNATIVE MEDICAL SYSTEMS | | | | |
| --- | --- | --- | --- | --- |
|  | Homeopathic remedies | □ | Naturopathic remedies | □ |
|  | Acupuncture | □ |  |  |
| BIOLOGICAL BASED THERAPIES | | | | |
| *Diets* | |  |  |  |
|  | Casein free | □ | Yeast free | □ |
|  | Gluten free | □ | Caffeine free | □ |
|  | Low glycemic diet | □ | Digestive enzymes | □ |
|  | No additive and preservatives | □ | Essential fatty acids liquid | □ |
|  | Sugar free | □ | Melatonin | □ |
| *Mineral supplements* | |  |  |  |
|  | Magnesium | □ | Selenium | □ |
|  | Calcium | □ |  | □ |
| *Mineral and Vitamin Supplements* | | |  |  |
|  | Magnesium/B6 | □ | Nutritional supplements | □ |
|  | Magnesium/B12 | □ | Omega 3 oil | □ |
| *Vitamin Supplements* | |  |  |  |
|  | Vitamin B | □ | Garlic oil | □ |
|  | Vitamin C | □ | Strawberry extract | □ |
|  | Echinacea | □ | Vitamin D | □ |
| MANIPULATIVE AND BODY-BASED THERAPIES | | | | |
|  | Body based relaxation therapies | □ | Sensory integration | □ |
|  | Chiropractic | □ | Therapeutic horseback riding | □ |
|  | Massage | □ |  |  |
| MIND-BODY AND PSYCHOLOGICAL THERAPIES | | | | |
|  | Music therapy | □ | Spiritual healing | □ |
|  | Counselling | □ | Play therapy | □ |
| OTHER (please specify): | | □ |  |  |

35. How close are you (on average) to your ASD-related COMPLEMENTARY/ALTERNATIVE (non-claimable) services (those that require travel)?

| - Less than 2 kilometres - 2-5 kilometres - 6-10 kilometres - 11-20 kilometres - 21-30 kilometres - Greater than 30 kilometres - Not Applicable |
| --- |

36. How many ASD-related COMPLEMENTARY/ALTERNATIVE (non-claimable) visits/appointments (those that require visits) does your child have in an average month?

- None
- Less than 2
- 2
- 3
- 4
- 5
- 6
- 7
- 8 or more

37. *What is the total cost of your child's ASD-related COMPLEMENTARY/ALTERNATIVE (non-claimable) treatment per year? (Please estimate)*

- *Less than $250*
- *$250 - 500*
- *$501 – 1,000*
- *$1,001 – 2,000*
- *$2,001 - 4,000*
- *$4,001 - 6,000*
- *More than $6,000*

**ABOUT OTHER SERVICES AND COSTS**

38. Does/did your child attend:

- Mainstream private school
- Mainstream public school
- Autism specific program in a mainstream school
- Education support centre/school
- Home schooling
- No school
- Other (please specify):

39. If your child diagnosed with ASD attends a mainstream school, was your choice of school influenced by their eligibility for aide time or funding?

- Yes
- No

40. If your child diagnosed with ASD attends a school or education support centre, approximately how many hours per week does your child receive aide/education support in the class room?

- No in-class support
- Less than 5 hours
- Between 5-10 hours
- Between 10-15 hours
- More than 15 hours

41. Do you access any of the following child-care services for your child diagnosed with ASD?

- After-school care
- Day care
- Baby-sitter in the evenings
- Baby-sitter on weekends
- Children’s residential respite
- In-home respite
- None

42. If you selected any options in the previous question, how many hours do you arrange in the following activities in a typical week?

| After-school care | Day care | Baby-sitter (evenings) | Baby-sitter (weekends) | In-home respit |
| --- | --- | --- | --- | --- |
| □ none | □ none | □ none | □ none | □ day only |
| □ less than 2 hours | □ less than 5 hours | □ less than 3 hours | □ less than 3 hours | □ overnight |
| □ 2-5 hours | □ 10 hours | □ 3-5 hours | □ 3-5 hours | □ weekends |
| □ 5-8 hours | □ 15 hours | □ 5-8 hours | □ 5-8 hours |  |
| □ 8-12 hours | □ >20 hours | □ More than 8 hours | □ More than 8 hours |  |

43. Do you arrange child-care for your other children due to the needs of your child diagnosed with an ASD?

- Yes

If yes, how many hours do you arrange in the following activities for your other children?

| After-school care | Day care | Baby-sitter  (evenings) | Baby-sitter  (weekends) |
| --- | --- | --- | --- |
| □ none | □ none | □ none | □ none |
| □ less than 2 hours | □ less than 5 hours | □ less than 3 hours | □ less than 3 hours |
| □ 2-5 hours | □ 10 hours | □ 3-5 hours | □ 3-5 hours |
| □ 5-8 hours | □ 15 hours | □ 5-8 hours | 5-8 hours |
| □ 8-12 hours | □ >20 hours | □ More than 8 hours | □ More than 8 hours |

- No

44. How difficult is it for you to find baby-sitters for your child with ASD?

- Not Difficult At All
- Somewhat Difficult
- Very Difficult
- Not Applicable

45. How often have you utilised respite care for your child or adult with ASD?

- Never
- Occasionally
- Weekly
- Fortnightly
- Monthly

**The next FOUR questions with an * are only relevant to those parents with adult sons/daughters diagnosed with an ASD. If you do not have an adult child with ASD, please proceed to Question 51.**

46. *****Has your ADULT son/daughter with ASD completed any tertiary studies?

- No
- Apprenticeship
- TAFE certificate
- University (undergraduate)
- University (postgraduate)

47. ***** If you answered yes to the previous question,how were these studies funded?

- Out of pocket
- Scholarship from the tertiary institution
- HECS or other government support
- Supported by private organisation

48. ***** What is the employment status of your ADULT child with ASD?

- Unemployed
- Volunteer work
- Part-time supported work
- Full-time supported work
- Part-time work
- Full-time work

49. ***** Where does your ADULT child with ASD live?

- At home with me
- In a group home
- In a care facility
- Semi-independently
- Completely independently

**ABOUT YOU AND YOUR FAMILY**

50. *What is the employment status of your household/s? (NB: ‘Parent’ refers to primary caregivers whether they are biological, step, foster or adoptive)*

- Both parents employed full time
- No parent employed
- One parent employed full time, one parent employed part time (half-time or more)
- One parent employed full time, one parent employed part time (less than half-time)
- One parent employed full time
- Both parents employed part time
- Single parent employed full time
- Single parent not employed
- Single parent employed part time (half-time or more)
- Single parent employed part time (less than half-time)

*51. How much has your child’s ASD diagnosis affected the employment status of your household?*

- Both parents must work less hours
- One parent (of a two-parent household) must work less hours
- Single parent must work less hours
- One parent cannot work at this time
- Both parents cannot work at this time
- Unaffected

*52. If your employment status has been affected, please estimate by how many hours in an average week your employment load has been reduced.*

- Not relevant
- Less than 7
- 7 – 14 hours
- 15 – 21 hours
- 22 – 28 hours
- 29 – 35 hours
- More than 35 hours

**ABOUT YOU**

53. What services do you currently access, or have you accessed in the past, for yourself? Please tick all that apply.

- Group Counselling/Support group
- Family Therapy/Counselling or couples therapy/counselling
- Individual Therapy/Counselling
- Respite Care for your children
- Parent Training Classes
  I do not access any services
- Other (please specify):

54. *How would you rate your family's overall strain/stress due to your child's ASD diagnosis?*

| No stress | Mild stress | Moderate  stress | Severe  stress | Very severe stress | Worst  possible stress |
| --- | --- | --- | --- | --- | --- |
| □ | □ | □ | □ | □ | □ |


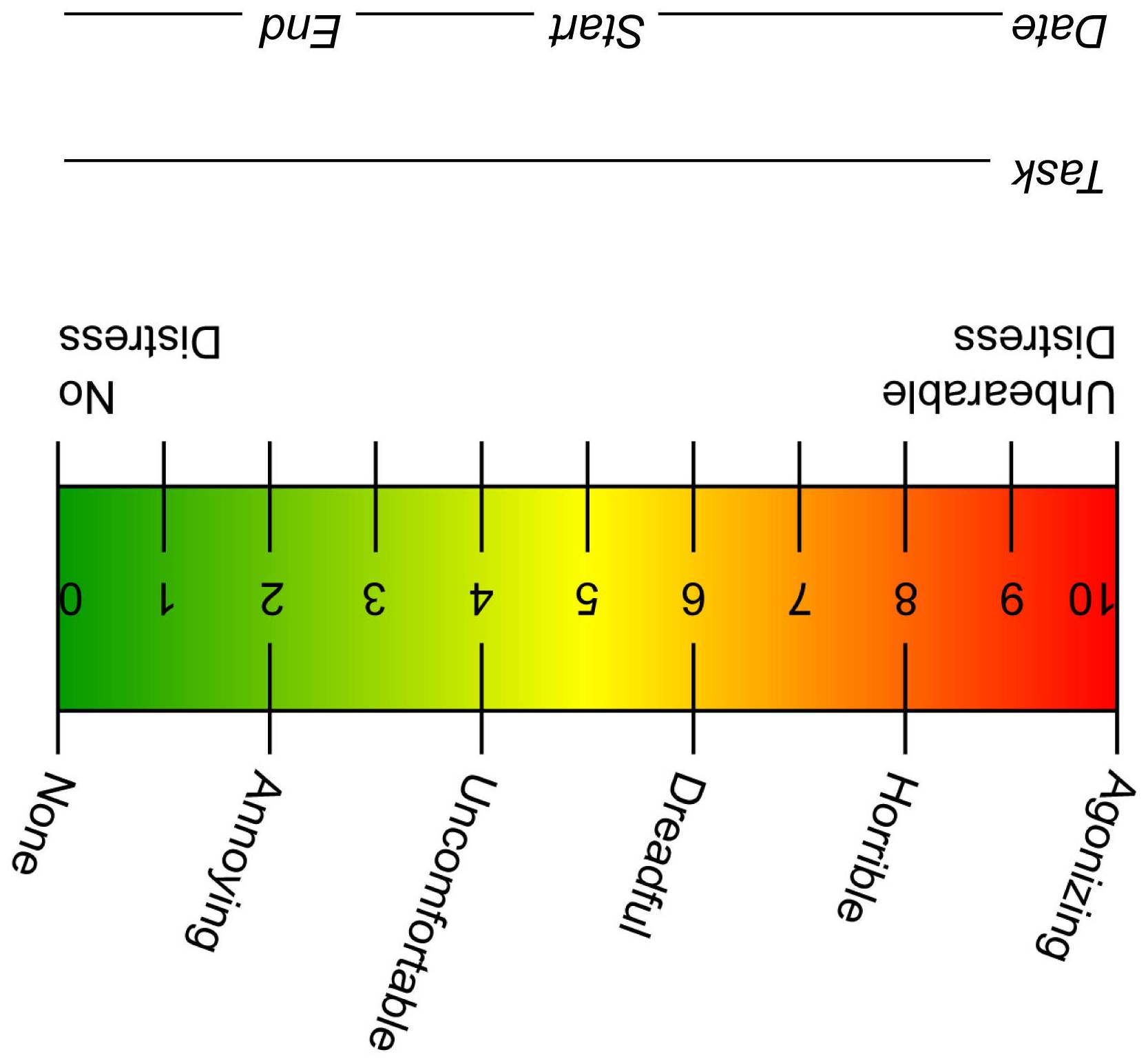


55. *How has your child's diagnosis of ASD affected YOUR relationship with your OTHER children?*

- A great positive impact on your relationship
- A slight positive impact on your relationship
- No impact on your relationship
- A slight negative impact on your relationship
- A great negative impact on your relationship
- Not Applicable (Only have one child)

56. *How has your child's diagnosisaffected YOUR relationship with your partner/co-parent?*

- A great positive impact on your relationship
- A slight positive impact on your relationship
- No impact on your relationship
- A slight negative impact on your relationship
- A great negative impact on your relationship
- Not Applicable (single parent)

57. How much of an impact has having a child with an ASD had on your ability to get out and socialize?

| No impact | Mild impact | Moderate  impact | Severe  impact | Very severe impact | Worst  possible impact |
| --- | --- | --- | --- | --- | --- |
| □ | □ | □ | □ | □ | □ |
| 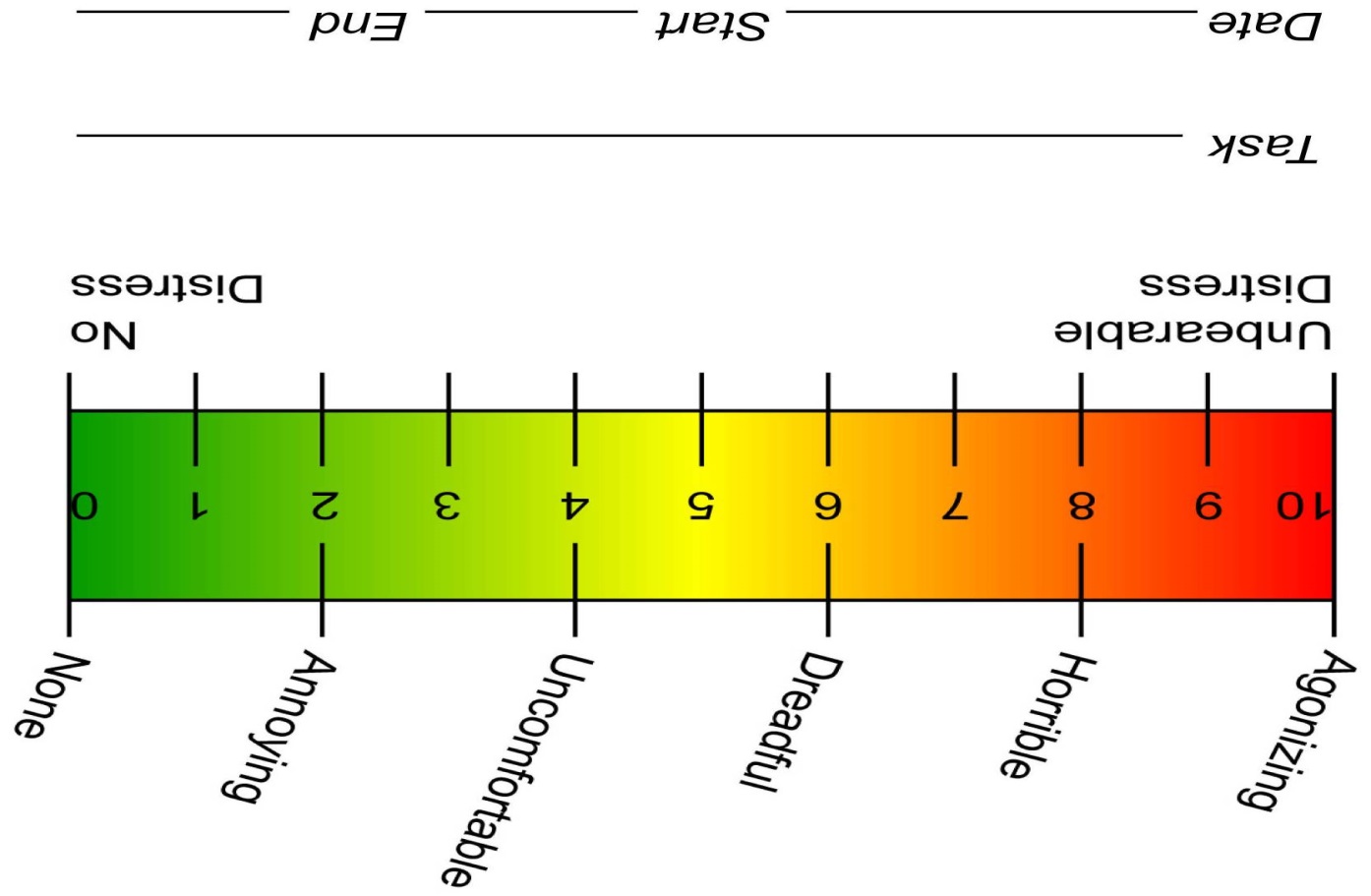 | | | | | |

58. Approximately how many hours are you able to participate in leisure activities per week?

59. How would you rate YOUR level of social support?

| Lots of social support | Some social support | A little social support | No social support |
| --- | --- | --- | --- |
| □ | □ | □ | □ |
| 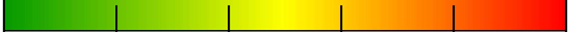 | | | |

60. Who do you receive support from? Tick all that apply

- My/our parents and/or siblings
- Extended family members
- Friends
- Other families with children diagnosed with ASD
- Support groups (either online or in person)
- Other (please specify):

ABOUT YOUR CHILD DIAGNOSED WITH ASD

61. How many hours per week does your child with ASD spend playing with friends (outside of school or other organised group activities)?

- None
- Up to 2 hours
- Up to 4 hours
- Up to 8 hours
- Up to 12 hours
- More than 12 hours

62. ASD-related intervention/behavioural therapy has NOT improved my child’s quality of life?

- Strongly Disagree
- Disagree
- Neutral
- Agree
- Strongly Agree
- Not Applicable

63. Has your child’s ASD-related intervention/behavioural therapy improved your family life?

- Yes, definitely
- Yes, somewhat
- I am neutral regarding this
- No, not at all
- Not Applicable

If you would like to explain your response, feel free to do so in the space on the following page:

64. Do you feel that earlier access to intervention may have led to more improvement in your child’s quality of life?

- Yes, definitely
- Yes, somewhat
- I am neutral regarding this
- No, not at all
- Not Applicable

If you would like to explain your response, feel free to do so in the space below:

ABOUT YOUR OTHER CHILDREN

65. What services do your other children (non-diagnosed) utilize?

- My child has no siblings
- The siblings do not utilize any services
- Family counselling
- Support group
- Individual counselling
- Other (please specify):

**ABOUT YOUR HOUSEHOLD**

66. *What is your current post code?*

67. Have you moved to a new home since your child was diagnosed with ASD? And if so why?

- No, we have not moved since my child was diagnosed with ASD
- Yes, because one or both caregivers changed location of employment
- Yes, in order to be closer to better treatment opportunities for my child
- Yes, other reason (Please explain)

68. *What is your household composition?*

- Two-parent
- Single parent
- Extended family (grandparents etc.)
- Two-parent plus Extended family
- Single parent plus Extended family
- Foster situation
- Other (please specify):

69. What is the mother's highest level of completed education?

- Completed year 10
- Completed year 12
- Completed certificate at TAFE (or similar)
- Apprenticeship
- Some university education, but did not complete
- Completed university undergraduate degree
- Completed university postgraduate degree

70. What is the father's highest level of completed education?

- Completed year 10
- Completed year 12
- Completed certificate at TAFE (or similar)
- Apprenticeship
- Some university education, but did not complete
- Completed university undergraduate degree
- Completed university postgraduate degree

71. What is your combined annual household income?

- Less than $25,000
- $25,000-50,000
- $50,000-75,000
- $75,000-100,000
- $100,000-125,000
- $125,000-150,000
- $150,000-200,000
- Greater than $200,000
- Unknown

72. Please estimate how much you have spent on home improvements/modifications to meet the needs of your child diagnosed with ASD since their initial diagnosis.

- Nothing
- Under $500
- $500 to $1000
- $1000- $2000
- $2000 - $5000
- $5000 - $10,000
- More than $10,000

The final question on the reverse of this page is a short checklist relating to your child’s behaviour and development. Thank you very much for your time in completing this questionnaire, your patience and contribution is greatly appreciated.

Checklist

73. Please indicate “Y” if the following applies/d to your child. Indicate “N” if the item does/did not apply to your child.

**1. Social interaction:**

____ Impairment in the use of multiple nonverbal behaviours, such as eye-to-eye gaze (e.g., eye contact), body posture, or gestures

____ Failure to develop peer relationships appropriate to developmental level (e.g., little to no interest in forming friendships or lack understanding of how to interact socially with others)

____ Lack of spontaneous seeking to share enjoyment, interest or achievements with others (e.g., not showing, bringing, or pointing out objects he/she finds interesting)

____ Lack of social or emotional reciprocity (e.g., not actively participating in social play or games, preferring solitary activities)

____ Rarely seeking or using others for comfort in times of stress or rarely offering comfort or affection to others in stress

**2. Communication:**

____ Delay in development or lack of spoken language (i.e., not accompanied by an attempt to communicate through alternative ways to communicate, such as gestures or mime)

____ In those with adequate speech, impairment to initiate or sustain conversations with others

____ Stereotyped and repetitive use of language or idiosyncratic/peculiar language (e.g., using words in a peculiar or odd way)

____ Lack of varied, spontaneous make-believe play (e.g., pretend/imaginary play) or socially imitative play (e.g., imitating adults) appropriate to developmental level

____ Lack of emotional response to others’ verbal or non-verbal communication

____ Lack of variation in the rhythm or emphasis of speech (e.g., speech is monotone or flat; without change)

____ Impaired use of gestures/nonverbal behaviour to aid spoken communication

**3. Restricted, repetitive and stereotyped patterns of behaviour, interest or activities:**

____ Preoccupation with one or more stereotyped and restricted patterns of interest with abnormal intensity or focus (e.g., few interests)

____ Inflexible attachment to specific, non-functional/essential routines or rituals

____Stereotyped and repetitive motor mannerisms (e.g., hand or finger flapping or twisting, or other complex whole-body movements such as rocking, dipping or swaying)

____ Persistent preoccupation with parts of objects (e.g., buttons, parts of the body)

____ Specific attachments to unusual objects (e.g., string)

____ Distress over changes in small, non-functional/essential details of the environment

____ Either overly-sensitive or under-sensitive to sensory input (e.g., sight, sound, touch, smell, taste, balance or body awareness)

**4. Developmental history**

_____ Delays or abnormal functioning in at least one of the previous areas (#1-3) was present prior to age of 3
